# Post-COVID-19 Consequences and Psychological Well-Being in Students: The Mediating Role of Trait Anxiety

**DOI:** 10.3390/bs16060996

**Published:** 2026-06-15

**Authors:** Sergey Malykh, Valeriia Demareva, Artem Malykh, Victoria I. Ismatullina, Timofey Adamovich, Pavel Kolyasnikov, Tatiana Tikhomirova

**Affiliations:** 1 Center for Interdisciplinary Research in the Educational Sciences, Russian Academy of Education, 119121 Moscow, Russia; malykhartem86@gmail.com (A.M.); victoria2686@gmail.com (V.I.I.); tadamovich11@gmail.com (T.A.); pavelkolyasnikov@gmail.com (P.K.); tikho@mail.ru (T.T.); 2 Cyberpsychology Department, Faculty of Social Sciences, Lobachevsky State University of Nizhny Novgorod, 603950 Nizhny Novgorod, Russia; valeriia.demareva@fsn.unn.ru

**Keywords:** post-COVID-19 consequences, psychological well-being, university students, trait anxiety, subjective happiness, life satisfaction, mediation analysis, long COVID

## Abstract

The long-term psychological consequences of COVID-19 remain insufficiently understood in student populations. This study examined the association between post-COVID-19 consequences and psychological functioning in university students, focusing on the mediating role of trait anxiety. A total of 7482 students aged 17 to 23 years completed an online survey assessing COVID-19 history, post-COVID-19 consequences, psychological well-being (WHO-5), subjective happiness (SHS), life satisfaction (SWLS), and trait anxiety (STAI). Participants were classified into three groups: no history of COVID-19, COVID-19 without post-COVID-19 consequences, and COVID-19 with post-COVID-19 consequences. Group differences were analyzed using ANOVA with Tukey post hoc tests, followed by regression and mediation analyses controlling for age and sex. Students reporting post-COVID-19 consequences showed higher trait anxiety and lower psychological well-being, subjective happiness, and life satisfaction than both comparison groups. Regression analyses indicated that poorer psychological functioning was associated specifically with post-COVID-19 consequences rather than COVID-19 history per se. Mediation analyses among previously infected students showed that trait anxiety statistically mediated these associations, accounting for 61% of the effect on psychological well-being, 84% on subjective happiness, and 68% on life satisfaction. These findings highlight trait anxiety as an important psychological factor statistically accounting for the association between post-COVID-19 consequences and reduced well-being.

## 1. Introduction

The coronavirus disease 2019 (COVID-19) pandemic has been a watershed event in global public health and mental health. Early data suggested that psychological distress surged across populations, with university students and emerging adults being particularly vulnerable. College students faced abrupt closures, transitions to remote learning, financial and familial stressors, and disruptions in developmental tasks related to autonomy, relationships, and career planning (Bersia et al., 2024). Several cross-sectional studies and meta-analyses during the first pandemic year reported pooled prevalence estimates of approximately 29% for anxiety and 23% for depression among university students (Ebrahim et al., 2022), substantially higher than pre-pandemic rates. Psychological difficulties persisted during later stages of the pandemic; repeated cross-sectional surveys found that moderate anxiety and depression remained elevated even after restrictions were lifted (Chen et al., 2024). Women and students with pre-existing mental health conditions were disproportionately affected (Bersia et al., 2024). These data highlight that university students are a high-risk group, and the pandemic accelerated a concerning trend toward increased mental health problems in emerging adults (Bersia et al., 2024; Chen et al., 2024).

Beyond acute pandemic stress, attention has shifted to the post-COVID-19 condition (or long COVID), defined by the World Health Organization as symptoms that emerge or persist three months after infection and last at least two months without alternative explanation. Long COVID is a multisystem condition characterized by a wide range of persistent symptoms affecting multiple domains of functioning. Prevalence estimates vary widely depending on methodology, population characteristics, and symptom definitions. Meta-analytic evidence suggests that a considerable proportion of individuals report ongoing symptoms, including fatigue (approximately 25–30%), anxiety (approximately 6–23%), and cognitive impairment (approximately 20%), although estimates may be considerably higher in some cohorts (Shen et al., 2025).

Cognitive dysfunction is increasingly recognized as one of the most persistent and disabling features of long COVID. Longitudinal studies indicate that cognitive complaints can emerge early after infection and may persist over time, with individuals reporting such symptoms in the first months after infection showing elevated risk of longer-term difficulties (Liu et al., 2023). In previously hospitalized COVID-19 survivors, self-reported symptoms such as brain fog, memory difficulties, and concentration problems have been documented during the first year after discharge, although prevalence tends to decline over time (Fernández-de-las-Peñas et al., 2023). Review evidence further suggests that impairments in attention, memory, processing speed, and executive functioning may persist for months after infection, with prevalence estimates often ranging from approximately 20% to 26% at 3–12 months (Popa et al., 2025). These cognitive sequelae may occur independently of mood symptoms (Popa et al., 2022) and, in some respects, resemble patterns of cognitive dysfunction observed in other medical conditions, including chemotherapy-related cognitive impairment (Velichkovsky et al., 2023).

In addition to cognitive sequelae, long COVID is also associated with substantial emotional and psychological consequences. Longitudinal evidence indicates that individuals with long COVID exhibit persistently elevated anxiety and depressive symptoms compared with those with short COVID, with differences remaining detectable up to 22 months after infection (Fancourt et al., 2023). Similarly, in a prospective cohort of patients hospitalized for severe COVID-19, anxiety symptoms were present in 33% of patients, depressive symptoms in 49%, and both perceived stress and cognitive dysfunction in 11% approximately two years after the acute illness (Rivas Nieto et al., 2026). These findings suggest that post-COVID-19 consequences may include persistent psychological distress, fatigue, and cognitive dysfunction (Bhargava et al., 2025; Taquet et al., 2022). Fatigue appears to be particularly burdensome; clinical data from a long COVID cohort indicate a high prevalence of clinically significant and severe fatigue, which was closely associated with depressive symptomatology (Bhargava et al., 2025). Several mechanisms have been proposed, including immune dysregulation, neuroinflammation, dysbiosis and hypothalamic–pituitary–adrenal axis dysfunction, suggesting that physical and psychological symptoms are intertwined.

Although long COVID research includes diverse populations, students and young adults have received limited attention. A cross-sectional study of Chinese medical students conducted after the first nationwide post-pandemic wave reported high rates of anxiety (37.8%), depression (39.3%), insomnia (28.3%), and post-traumatic stress symptoms (29.5%). Students with rural childhood or current residence also reported significantly higher levels of self-perceived cognitive symptoms than students from urban settings (Cheng et al., 2023). These findings illustrate that even without confirmed infection, pandemic-related stressors may produce mental health symptoms, but they do not address post-COVID-19 consequences. Empirical evidence on how lingering post-infection symptoms affect students’ daily functioning and quality of life remains limited. In a Swedish cross-sectional study of individuals with post-COVID-19 condition, 77% reported low overall life satisfaction, and 87% perceived their life satisfaction as deteriorated compared with the pre-COVID-19 period; 98% attributed this change at least partly to COVID-19. Poor work ability was the strongest correlate of low life satisfaction, while reduced aerobic capacity and both mental and physical fatigue also increased the odds of poorer outcomes (Ekstrand et al., 2022). These findings suggest that post-COVID-19 symptoms may substantially impair multiple domains of daily functioning and overall subjective well-being.

Psychological well-being is a multidimensional construct encompassing hedonic and eudaimonic aspects such as positive affect, life satisfaction, and purpose. Three widely used instruments capture different facets of well-being and are employed in the present study. The WHO-5 Well-Being Index is a five-item scale assessing positive mood and vitality. Systematic reviews describe it as one of the most widely used questionnaires for subjective psychological well-being; it is brief, positively worded, and available in many languages (Domenech et al., 2025). Cross-cultural validation studies during the pandemic found Cronbach’s alpha values between 0.81 and 0.90 and strong correlations with psychological quality-of-life measures, supporting its unidimensional structure and psychometric soundness (Lara-Cabrera et al., 2022). The Subjective Happiness Scale (SHS) is a four-item instrument providing a global measure of subjective happiness; it has high internal consistency and test–retest reliability across diverse populations (Lyubomirsky & Lepper, 1999) and demonstrates moderate-to-strong associations with related constructs such as resilience and self-efficacy (Basnet et al., 2025). The Satisfaction with Life Scale (SWLS) measures the cognitive component of subjective well-being, assessing global life satisfaction independent of affective states. The original validation reported high internal consistency and temporal reliability and noted that scores correlate with other well-being measures and personality characteristics across age groups (Diener et al., 1985). Together, these scales provide a comprehensive assessment of well-being, happiness, and life satisfaction.

Anxiety exists as both a transient state and a stable personality trait. Trait anxiety reflects an enduring tendency to perceive situations as threatening and to respond with prolonged worry and physiological arousal. The State–Trait Anxiety Inventory (STAI) is widely used to distinguish trait from state anxiety. Trait anxiety is regarded as a risk factor for mental health problems; individuals with high trait anxiety are more sensitive to threats, prone to intrusive thoughts and rumination, and may have difficulty disengaging from negative information (Cécillon et al., 2024). According to attention control theory, anxiety is associated with reduced processing efficiency due to the allocation of cognitive resources toward threat-related information (Eysenck et al., 2007). More recent empirical work supports this framework, demonstrating associations between trait anxiety, executive functioning, and cognitive control processes (Cécillon et al., 2024). Trait anxiety has been consistently associated with lower well-being and increased vulnerability to psychological distress (Akat, 2025).

Emerging evidence suggests that anxiety may persist after COVID-19 infection and could be a key mechanism linking post-COVID-19 symptoms to well-being. Several mechanisms have been proposed, including immune dysregulation, neuroinflammation, and microbiome alterations, which may contribute to long-term changes in neurobiological and stress-regulation systems and potentially promote increased vulnerability to anxiety (Büttiker et al., 2021). Longitudinal evidence indicates persistently elevated anxiety and depressive symptoms in individuals with long COVID (Fancourt et al., 2023). Post-COVID-19 cognitive impairments may exacerbate anxiety by reducing processing capacity and increasing uncertainty about recovery. Evidence from developmental samples suggests that individuals with higher trait anxiety show attentional bias toward threat-related information, although this relationship may vary depending on age and contextual factors (Zhang, 2023). During the pandemic, trait anxiety was linked to higher fear of COVID-19, and resilience mediated approximately 35% of the association between trait anxiety and fear in Brazilian university students (Gonçalves et al., 2021). Pre-pandemic research among university students indicates that trait anxiety may already be highly prevalent in this population. In one survey conducted in 2019, only 7.4% of students showed normal levels of trait anxiety, whereas 75% were classified as having elevated trait anxiety (Dias Lopes et al., 2020). In the same study, higher trait anxiety was closely associated with generalized anxiety and lower well-being. However, research has not yet examined whether trait anxiety mediates the effect of post-COVID-19 symptoms on well-being.

Most studies on student mental health during the COVID-19 pandemic have focused on immediate stressors such as lockdowns, fear of infection, and disruption of academic routines (Bersia et al., 2024). While this literature demonstrates a high prevalence of anxiety and depression and highlights protective factors like resilience and adaptive emotion regulation (Bersia et al., 2024; Chen et al., 2024), it does not address post-infection sequelae. There is a paucity of research on students who have recovered from COVID-19 and are experiencing lingering physical, cognitive, or emotional symptoms. Long COVID studies often involve general adult populations or hospital cohorts (Shen et al., 2025; Fancourt et al., 2023), making it difficult to generalize findings to university students. Moreover, the mechanisms linking post-COVID-19 consequences to psychological well-being remain insufficiently understood. Trait anxiety, a stable vulnerability factor, may be an important mediator given its associations with cognitive resource depletion, negative affect, generalized anxiety, and poorer well-being (Cécillon et al., 2024; Akat, 2025). However, no study to date has empirically tested whether trait anxiety mediates the relationship between post-COVID-19 symptoms and well-being or life satisfaction among students.

To address these gaps, the present study examines the association between post-COVID-19 consequences and psychological functioning among university students. Using a cross-sectional design, we analyzed a large sample of students, distinguishing between individuals with no history of COVID-19, those who had COVID-19 without post-COVID-19 consequences, and those who reported post-COVID-19 consequences. Participants provided information about their COVID-19 experience and the presence of persistent post-COVID-19 symptoms. Importantly, this design allows for distinguishing between the effects of COVID-19 infection per se and the effects of post-COVID-19 consequences.

Psychological functioning was assessed using three complementary indicators: the WHO-5 Well-Being Index, the Subjective Happiness Scale, and the Satisfaction With Life Scale, capturing positive affect, global happiness, and cognitive evaluations of life satisfaction, respectively (Domenech et al., 2025; Lyubomirsky & Lepper, 1999; Diener et al., 1985). Trait anxiety was measured using the trait form of the State–Trait Anxiety Inventory (Spielberger, 1989).

Based on previous research, we hypothesized that students reporting post-COVID-19 consequences would demonstrate poorer psychological functioning compared to both students without a history of COVID-19 and those who had COVID-19 without consequences. In addition, we hypothesized that trait anxiety would statistically mediate the association between post-COVID-19 consequences and psychological outcomes, such that the presence of post-COVID-19 consequences would be associated with higher trait anxiety, which in turn would be linked to lower well-being, happiness, and life satisfaction.

By identifying trait anxiety as a potential explanatory factor, the present study seeks to clarify how post-COVID-19 consequences are linked to reduced psychological functioning in students and to inform the development of targeted psychological interventions for this vulnerable population.

## 2. Materials and Methods

### 2.1. Participants

The sample consisted of 7482 university students recruited from 30 universities across the Russian Federation, covering all federal districts. Data were collected using a structured online survey assessing psychological well-being, subjective happiness, life satisfaction, trait anxiety, and COVID-19-related experiences.

After data cleaning, participants aged 17 to 23 years were retained for analysis (M = 18.51, SD = 0.96). Sex was coded as a binary variable (1 = male, 2 = female). The sample consisted of 2694 males (36.0%) and 4788 females (64.0%).

Among the participants, 3537 individuals (47.3%) reported having had COVID-19, whereas 3945 (52.7%) reported no history of COVID-19. Based on self-reported information about post-COVID-19 consequences, 1861 participants (24.9% of the total sample) indicated experiencing at least one persistent consequence following infection. This corresponds to approximately 52.6% of participants who reported a history of COVID-19.

All procedures were conducted in accordance with the ethical standards of the 1964 Declaration of Helsinki and its later amendments. Written informed consent was obtained from all adult participants prior to participation. For participants younger than 18 years, written informed consent was obtained from their legal guardians, and assent was obtained from the participants themselves. All participants were informed about the purpose of the study and participated voluntarily. The study protocol was approved by the Ethics Committee of the Psychological Institute of the Russian Academy of Education (Protocol No. 2020/4-1, 2 April 2020).

### 2.2. Measures

#### 2.2.1. Psychological Well-Being

Psychological well-being was assessed using the World Health Organization Five Well-Being Index (WHO-5). The WHO-5 is a five-item self-report measure assessing positive mood, vitality, and general well-being. Scores range from 0 to 100, with higher scores indicating greater psychological well-being.

#### 2.2.2. Subjective Happiness

Subjective happiness was measured using the Subjective Happiness Scale (SHS) developed by Lyubomirsky and Lepper. The SHS consists of four items assessing global subjective happiness. Higher scores indicate greater perceived happiness.

#### 2.2.3. Life Satisfaction

Life satisfaction was assessed using the Satisfaction with Life Scale (SWLS) developed by Diener and colleagues. The SWLS consists of five items measuring global cognitive judgments of life satisfaction. Higher scores indicate greater life satisfaction.

#### 2.2.4. Trait Anxiety

Trait anxiety was measured using the Trait subscale of the State–Trait Anxiety Inventory (STAI). The STAI is a widely used instrument assessing stable individual differences in anxiety proneness. Higher scores indicate higher levels of trait anxiety.

#### 2.2.5. COVID-19 Variables

Participants reported whether they had previously been diagnosed with COVID-19. In addition, participants indicated the presence of specific post-COVID-19 consequences, including decreased mood, increased anxiety, memory difficulties, concentration problems, and other self-reported symptoms.

Based on these responses, a binary variable indicating the presence of post-COVID-19 consequences was created (0 = no reported consequences; 1 = at least one reported consequence).

In the present study, the term “post-COVID consequences” refers to self-reported persistent difficulties that participants attributed to previous COVID-19 infection. This operational definition is broader than the clinical definition of long COVID and does not imply a medically verified diagnosis. Because information on symptom duration, frequency, and severity was not available, this variable should be interpreted as an indicator of perceived post-COVID-19 consequences rather than clinically confirmed long COVID.

To capture differences between infection status and post-COVID-19 outcomes, a three-level categorical variable was constructed:

- No history of COVID-19;
- COVID-19 without post-COVID-19 consequences;
- COVID-19 with post-COVID-19 consequences.

This variable was used as the primary predictor in group-based analyses.

Internal consistency in the present sample was good to excellent—Cronbach’s α = 0.868 for WHO-5, α = 0.803 for SHS, α = 0.867 for SWLS, and α = 0.909 for STAI.

### 2.3. Procedure

Data collection was conducted in supervised classroom settings during regular academic hours (9:00–13:00). Participants completed the online questionnaire individually using institutional computers.

The survey included demographic questions, COVID-19-related items, and standardized psychological measures (WHO-5, SHS, SWLS, STAI). The total duration of participation was approximately 15–20 min. Participants were instructed to read each item carefully and respond as accurately as possible. Participation was voluntary, and no time limits were imposed.

### 2.4. Statistical Analysis

All statistical analyses were conducted using R (version 4.4.1; R Core Team, 2024) in the RStudio environment (version 2024.04.2 Build 764).

Descriptive statistics were computed for all study variables. Group differences across the three COVID-19 groups were examined using one-way analysis of variance (ANOVA), followed by Tukey post hoc tests for pairwise comparisons.

To examine associations between COVID-19 group membership and psychological outcomes, a series of linear regression analyses were conducted with psychological well-being (WHO-5), subjective happiness (SHS), and life satisfaction (SWLS) as dependent variables. Age and sex were included as control variables in all models.

Additional analyses were conducted in the subsample of participants with a history of COVID-19 to examine differences between individuals with and without post-COVID-19 consequences.

To test the proposed psychological mechanism, mediation analyses were conducted in the subsample of participants with a history of COVID-19. Trait anxiety (STAI) was specified as a mediator of the relationship between post-COVID-19 consequences and psychological outcomes. Indirect effects were estimated using nonparametric bootstrap procedures with 5000 resamples, implemented via the *mediation* package (version 4.5.1; Tingley et al., 2014). Age and sex were included as covariates in both mediator and outcome models.

All analyses were conducted using available-case data. Observations with missing values for variables included in a given model were excluded from that analysis. Statistical significance was set at *p* < 0.05.

## 3. Results

### 3.1. Group Differences

Descriptive statistics for psychological variables across the three COVID-19 groups are presented in Table 1.

One-way analyses of variance revealed significant group differences across all psychological variables, including trait anxiety, F = 120.0, psychological well-being, F = 110.2, subjective happiness, F = 21.6, and life satisfaction, F = 38.6 (all *p* < 0.001). Students reporting COVID-19 with post-COVID-19 consequences demonstrated the highest trait anxiety scores (M = 44.7, SD = 10.7) compared to both students without a history of COVID-19 (M = 40.9, SD = 10.8) and students who had COVID-19 without consequences (M = 39.3, SD = 10.6). The same group also showed the lowest psychological well-being (WHO-5: M = 57.1, SD = 20.3), subjective happiness (SHS: M = 17.8, SD = 3.4), and life satisfaction (SWLS: M = 22.4, SD = 6.1). By contrast, students who had experienced COVID-19 without post-COVID-19 consequences demonstrated psychological functioning comparable to or slightly better than the no-COVID-19 group, particularly in life satisfaction (M = 23.8 vs. 23.1) and psychological well-being (M = 66.0 vs. 64.6). Post hoc Tukey comparisons confirmed that the group reporting post-COVID-19 consequences differed significantly from both comparison groups across all outcomes. Effect sizes were small to moderate, with η^2^ = 0.03 for trait anxiety, η^2^ = 0.03 for psychological well-being, η^2^ = 0.006 for subjective happiness, and η^2^ = 0.01 for life satisfaction.

Overall, the most unfavorable psychological profile was observed in the group reporting post-COVID-19 consequences.

### 3.2. Regression Analysis

To further examine the association between COVID-19 status and psychological functioning, a series of linear regression analyses were conducted with the three-group COVID-19 variable as the predictor and psychological indicators as dependent variables. Age and sex were included as control variables in all models. The reference category was the group with no history of COVID-19. Table 2 summarizes the regression coefficients for all psychological outcomes.

Regression analyses showed that students who had COVID-19 without post-COVID-19 consequences did not differ substantially from the no-COVID-19 group in psychological well-being or subjective happiness, although they showed slightly lower trait anxiety and slightly higher life satisfaction. In contrast, students reporting COVID-19 with post-COVID-19 consequences demonstrated significantly higher trait anxiety and significantly lower psychological well-being, subjective happiness, and life satisfaction relative to the no-COVID-19 group.

These results suggest that poorer psychological functioning is associated not with a history of COVID-19 per se but specifically with the presence of post-COVID-19 consequences. The models explained 5.8% of the variance in trait anxiety, 4.1% in psychological well-being, 0.7% in subjective happiness, and 1.3% in life satisfaction.

### 3.3. Additional Comparisons Within Students with a History of COVID-19

To clarify whether post-COVID-19 consequences were associated with poorer outcomes among students who had experienced COVID-19, additional regression analyses were conducted in the subsample of infected participants only. In these models, students with COVID-19 but no consequences served as the reference group.

Across all outcomes, students reporting post-COVID-19 consequences demonstrated significantly poorer psychological functioning than students who had COVID-19 without consequences. Specifically, post-COVID-19 consequences predicted lower psychological well-being (β = −8.31, SE = 0.69, *t* = −12.00, *p* < 0.001), lower subjective happiness (β = −0.69, SE = 0.11, *t* = −6.04, *p* < 0.001), lower life satisfaction (β = −1.96, SE = 0.22, *t* = −9.10, *p* < 0.001), and higher trait anxiety (β = 4.74, SE = 0.37, *t* = 12.83, *p* < 0.001), controlling for age and sex.

These findings indicate that, among students with a history of COVID-19, the presence of post-COVID-19 consequences is robustly associated with poorer psychological adjustment. In the subsample of previously infected students, model fit indices showed a similar pattern. The models explained 8.4% of the variance in trait anxiety, 5.2% in psychological well-being, 1.3% in subjective happiness, and 2.6% in life satisfaction.

### 3.4. Mediation Analysis Among Students with a History of COVID-19

To test whether trait anxiety mediated the relationship between post-COVID-19 consequences and psychological functioning among students who had experienced COVID-19, mediation analyses were conducted in the infected subsample only. Age and sex were included as control variables in both mediator and outcome models. Indirect effects were estimated using nonparametric bootstrap procedures with 5000 resamples.

Trait anxiety significantly mediated the relationship between post-COVID-19 consequences and all three psychological outcomes (Table 3).

For psychological well-being (WHO-5), the indirect effect through anxiety was significant (ACME = −5.25, *p* < 0.001), accounting for approximately 61% of the total effect. For subjective happiness, the indirect effect was also significant (ACME = −0.58, *p* < 0.001), whereas the direct effect did not reach statistical significance (*p* = 0.299), suggesting that the association between post-COVID-19 consequences and subjective happiness was largely mediated by increased anxiety. For life satisfaction, trait anxiety again showed a significant indirect effect (ACME = −1.34, *p* < 0.001), accounting for approximately 68% of the total effect.

Taken together, these findings indicate that post-COVID-19 consequences are associated with poorer psychological functioning in students and that trait anxiety represents a substantial psychological mechanism linking post-COVID-19 consequences to lower well-being, happiness, and life satisfaction. The outcome models that included trait anxiety showed substantially higher explanatory power, accounting for 37.3% of the variance in psychological well-being, 16.3% in subjective happiness, and 24.7% in life satisfaction.

## 4. Discussion

The present study examined the association between post-COVID-19 consequences and psychological functioning in university students, with a particular focus on the mediating role of trait anxiety. Three key findings emerged, which are consistent with the study hypotheses. First, students reporting post-COVID-19 consequences demonstrated consistently higher levels of trait anxiety and lower psychological well-being, subjective happiness, and life satisfaction compared to both students without a history of COVID-19 and those who had COVID-19 without residual consequences. Second, these associations remained robust after controlling for age and sex. Third, and most importantly, trait anxiety accounted for a substantial proportion of the relationship between post-COVID-19 consequences and all indicators of psychological functioning, highlighting its role as a central psychological mechanism.

These findings align with the broader literature indicating that the impact of COVID-19 extends beyond the acute phase of infection and involves persistent psychological and cognitive difficulties (Fancourt et al., 2023; Shen et al., 2025; Rivas Nieto et al., 2026). However, the present study advances this research in two important ways. First, it differentiates between COVID-19 infection per se and post-COVID-19 consequences, demonstrating that reduced psychological functioning is not associated with having had COVID-19 alone, but specifically with the presence of lingering symptoms. Notably, students who had COVID-19 but did not report post-COVID-19 consequences did not differ—and in some cases showed slightly better outcomes—from those who had never been infected. This distinction is rarely made in prior research and suggests that post-COVID-19 consequences, rather than infection history itself, represent the critical risk factor for reduced well-being. Second, the study moves beyond descriptive associations by identifying a theoretically grounded statistical pathway linking post-COVID-19 consequences to psychological outcomes.

The pattern of group differences suggests that post-COVID-19 consequences are associated with a generalized reduction in psychological functioning rather than with isolated deficits. Lower scores were observed across all three indicators of well-being, encompassing both affective (WHO-5, SHS) and cognitive–evaluative (SWLS) components. At the same time, differences in effect sizes provide important nuance. The largest absolute differences were observed for psychological well-being (WHO-5), which reflects daily vitality and positive affect. However, the proportion of the effect mediated by trait anxiety was lower for WHO-5 than for subjective happiness and life satisfaction. This pattern suggests that while anxiety plays a substantial role, reductions in vitality and positive mood may also be associated with more direct physiological or somatic aspects of post-COVID-19 consequences, such as fatigue, reduced energy, and cognitive strain. This interpretation is consistent with previous findings indicating that fatigue and cognitive difficulties are among the most prominent features of post-COVID-19 condition (Bhargava et al., 2025; Ekstrand et al., 2022).

The mediation analyses provide the central theoretical contribution of the study. Trait anxiety statistically accounted for more than half of the association between post-COVID-19 consequences and psychological well-being, as well as 84% of the association with subjective happiness and 68% of the association with life satisfaction. For subjective happiness, the direct effect became non-significant after accounting for anxiety, indicating full mediation. For psychological well-being and life satisfaction, direct effects remained significant, indicating partial mediation. Taken together, these results largely support the proposed hypotheses, indicating that trait anxiety represents an important psychological mechanism linking post-COVID-19 consequences to reduced psychological functioning.

The relatively low explanatory power observed for subjective happiness and life satisfaction is not unexpected. Both constructs are multidetermined and influenced by numerous factors that were not assessed in the present study, including academic stress, socioeconomic conditions, social support, personality characteristics, and previous mental health history. Accordingly, post-COVID-19 consequences appear to represent only one of several contributors to these broader indicators of psychological functioning.

From a theoretical perspective, this pattern is highly plausible. Trait anxiety reflects a stable tendency to perceive situations as uncertain or threatening and to allocate attentional and cognitive resources toward potential risks. In the context of post-COVID-19 consequences, such a disposition may amplify concerns about persistent symptoms, academic performance, and future health. Cognitive complaints and fatigue may further increase uncertainty and reduce perceived control, thereby reinforcing anxious appraisal processes. In turn, elevated anxiety may be associated with poorer well-being through increased rumination, attentional bias toward negative information, and reduced engagement with rewarding or meaningful activities. Within the framework of attention control and resource allocation theories, anxiety can be understood as consuming cognitive and emotional resources that would otherwise support adaptive functioning (Cécillon et al., 2024). The present findings are consistent with this perspective and suggest that the observed pattern may involve both direct associations and indirect associations through heightened anxious vulnerability.

The results are particularly relevant in the context of student mental health. University students represent a population already characterized by elevated vulnerability to anxiety and affective disturbances due to developmental, academic, and social demands. Within this context, persistent post-COVID-19 symptoms may act as an additional burden, exacerbating existing vulnerabilities. Difficulties with concentration, memory, and energy may directly interfere with academic performance, while increased anxiety may further compromise coping and self-regulation. Importantly, the findings suggest that post-pandemic student well-being should not be viewed solely in terms of acute stressors such as lockdowns or fear of infection but also in terms of lingering post-infection consequences that may persist and interact with individual vulnerability factors.

An important strength of this study is the use of multiple indicators of psychological functioning. By assessing well-being, subjective happiness, and life satisfaction, the study captures both affective and cognitive dimensions of functioning. The differential pattern of mediation across these indicators provides additional insight into underlying mechanisms. Specifically, anxiety appears to play a particularly strong role in shaping global evaluations of life and happiness, whereas reductions in vitality and positive affect may also reflect more direct physiological influences. This differentiation would not be possible with a single outcome measure and highlights the importance of a multidimensional approach to well-being.

The findings also have practical implications. Given the substantial mediating role of trait anxiety, interventions targeting anxiety may be particularly effective for students experiencing post-COVID-19 consequences. Screening procedures in educational settings could benefit from simultaneously assessing post-COVID-19 symptoms and anxiety levels. Students reporting persistent symptoms may require not only medical monitoring but also psychological support aimed at reducing worry, improving tolerance of uncertainty, and enhancing attentional control and emotion regulation. Low-threshold interventions, including psychoeducation and cognitive–behavioral strategies, may help mitigate the impact of anxiety on daily functioning. More broadly, the findings suggest that post-COVID-19 consequences should be recognized as a factor affecting student mental health and integrated into university support systems.

Several limitations should be considered. First, the cross-sectional design precludes causal inference. Although the mediation model is theoretically grounded, mediation effects estimated from cross-sectional data should not be interpreted as evidence of causal pathways or temporal ordering among variables. It is possible that higher anxiety predisposes individuals to perceive or report more post-COVID-19 consequences, or that bidirectional relationships exist. Longitudinal studies are required to clarify causal pathways. Second, post-COVID-19 consequences were assessed via self-report and were not clinically verified, meaning that the results reflect perceived rather than medically confirmed conditions. This consideration is particularly important given that 52.6% of previously infected participants in the present sample reported at least one post-COVID-19 consequence, a proportion higher than some population-based estimates reported in the literature. This discrepancy may reflect the broad self-report definition used in the present study, where the presence of any one symptom was sufficient for classification, as well as the characteristics of a student sample, in which symptom awareness and willingness to report lingering difficulties may be relatively high. Third, all variables were measured using self-report instruments, raising the possibility of shared method variance. Fourth, the sample was restricted to university students aged 17–23 years, limiting generalizability to other populations. Fifth, the sample included a higher proportion of female participants (64.0%) than male participants (36.0%). Although sex was included as a covariate in all regression and mediation models, this imbalance may have influenced the overall levels of anxiety and well-being, given known sex differences in psychological distress. Finally, although age and sex were controlled, other potentially relevant variables—such as socioeconomic status, prior mental health history, academic stress, and social support—were not included.

The relatively low explanatory power observed for subjective happiness and life satisfaction suggests that these outcomes are influenced by numerous factors beyond post-COVID-19 consequences. Variables such as academic stress, socioeconomic status, prior mental health difficulties, personality characteristics, social support, and family context were not assessed in the present study and may account for additional variance in these indicators.

Future research should address these limitations by employing longitudinal designs (Servier et al., 2023) and more detailed assessments of post-COVID-19 symptom profiles. Differentiating between cognitive, emotional, and fatigue-related consequences may provide a more nuanced understanding of their psychological impact. In addition, examining moderating factors such as resilience, coping strategies, and social support could help identify which students are most vulnerable and which factors buffer against negative outcomes.

In summary, the present study demonstrates that post-COVID-19 consequences are associated with reduced psychological functioning in university students and identifies trait anxiety as a key mechanism underlying this relationship. These findings highlight the importance of considering both residual post-infection symptoms and individual vulnerability factors in understanding student well-being in the post-pandemic context.

## 5. Conclusions

The present study provides evidence that post-COVID-19 consequences, rather than COVID-19 infection per se, are associated with reduced psychological functioning in university students. Students reporting residual post-COVID-19 symptoms demonstrated lower psychological well-being, subjective happiness, and life satisfaction, even after accounting for age and sex. Crucially, trait anxiety emerged as a substantial mediator, explaining a large proportion of these associations.

These findings suggest that the psychological burden of post-COVID-19 consequences in university students is not limited to lingering symptoms themselves but is closely associated with heightened anxious vulnerability. Trait anxiety appears to be an important psychological factor associated with this relationship through which post-COVID-19 consequences may be associated with reduced functioning and diminished well-being.

Taken together, the results highlight the importance of integrating both symptom-related and psychological factors in understanding post-pandemic student well-being. Future longitudinal research is needed to clarify the temporal dynamics of these associations and to determine whether anxiety-focused interventions may help reduce the longer-term psychological burden of post-COVID-19 consequences in student populations.

## Figures and Tables

**Table 1:** Psychological characteristics of participants across three COVID-19 groups.

Variable	No COVID-19 (*n* = 3945)	COVID-19 Without Consequences (*n* = 1676)	COVID-19 With Consequences (*n* = 1861)	F
STAI (trait anxiety)	40.9 (10.8)	39.3 (10.6)	44.7 (10.7)	120.0
WHO-5 (psychological well-being)	64.6 (20.3)	66.0 (20.1)	57.1 (20.3)	110.2
SHS (subjective happiness)	18.3 (3.3)	18.4 (3.3)	17.8 (3.4)	21.6
SWLS (life satisfaction)	23.1 (6.5)	23.8 (6.2)	22.4 (6.1)	38.6

Values are presented as M (SD). Higher scores on STAI indicate higher trait anxiety, whereas higher scores on WHO-5, SHS, and SWLS indicate higher psychological well-being, subjective happiness, and life satisfaction.

**Table 2:** Regression models predicting psychological indicators from COVID-19 group membership (reference group: No COVID-19).

Outcome Variable	Predictor	β	SE	*t*	*p*
STAI	COVID-19 without consequences	−1.43	0.32	−4.52	<0.001
	COVID-19 with consequences	3.28	0.31	10.71	<0.001
	Age	0.35	0.13	2.67	0.008
	Sex	3.69	0.27	13.90	<0.001
WHO-5	COVID-19 without consequences	1.13	0.59	1.93	0.054
	COVID-19 with consequences	−6.86	0.57	−12.03	<0.001
	Age	−0.13	0.24	−0.52	0.603
	Sex	−4.76	0.49	−9.69	<0.001
SHS	COVID-19 without consequences	0.13	0.10	1.36	0.174
	COVID-19 with consequences	−0.55	0.09	−5.92	<0.001
	Age	−0.10	0.04	−2.44	0.015
	Sex	0.16	0.08	1.96	0.050
SWLS	COVID-19 without consequences	0.69	0.19	3.68	<0.001
	COVID-19 with consequences	−1.26	0.18	−6.93	<0.001
	Age	−0.24	0.08	−3.08	0.002
	Sex	0.56	0.16	3.54	<0.001

**Table 3:** Mediation analysis among students with a history of COVID-19: post-COVID-19 consequences, trait anxiety, and psychological outcomes.

Outcome Variable	Indirect Effect (ACME)	Direct Effect (ADE)	Total Effect	Proportion Mediated
WHO-5 (psychological well-being)	−5.25 *** (−6.10, −4.43)	−3.31 *** (−4.46, −2.16)	−8.56 *** (−9.96, −7.21)	0.61
SHS (subjective happiness)	−0.58 *** (−0.68, −0.48)	−0.11 (−0.32, 0.10)	−0.69 *** (−0.91, −0.46)	0.84
SWLS (life satisfaction)	−1.34 *** (−1.56, −1.11)	−0.64 ** (−1.02, −0.26)	−1.97 *** (−2.41, −1.56)	0.68

Values represent unstandardized coefficients with 95% bootstrap confidence intervals (5000 resamples). ACME = indirect effect through trait anxiety; ADE = direct effect of post-COVID-19 consequences on the outcome variable. *** *p* < 0.001, ** *p* < 0.01.

## Data Availability

The data presented in this study are available on request from the corresponding author.
